# Ligand‐Induced Electronic Response Enables Predictive QM/MM Simulations

**DOI:** 10.1002/advs.202519137

**Published:** 2025-12-22

**Authors:** Nichika Ozawa, Nahoko Kuroki, Hirotoshi Mori

**Affiliations:** ^1^ Department of Applied Chemistry Faculty of Science and Engineering Chuo University Bunkyo‐ku Tokyo Japan; ^2^ Faculty of Core Research Natural Science Division Ochanomizu University Bunkyo‐ku Tokyo Japan

**Keywords:** hybrid quantum/classical method (QM/MM), ligand‐induced electronic response, rational multiscale simulation, semiempirical fragment molecular orbital (FMO)‐guided active site definition

## Abstract

Predictive modeling of large molecular systems demands methods that combine quantum accuracy with scalability. Although hybrid quantum mechanics/molecular mechanics (QM/MM) simulations offer such a framework, their predictive power has been limited by the subjective and system‐specific definition of the QM region. Here, we present an electronically informed protocol that objectively defines QM regions from ligand‐induced orbital shifts and charge‐redistribution, extracted in a single semiempirical fragment molecular orbital (FMO) calculation. Validated on both zeolite–guest and enzyme–inhibitor complexes, the method achieves chemical accuracy (within ∼1–2 kcal/mol on binding energies) while substantially reducing computational cost at the DFTB level. This cross‐domain strategy reframes QM/MM as a transferable design principle, bridging solid‐state catalysis and quantum biochemistry, and thereby providing a practical platform for predictive molecular engineering across diverse disciplines.

## Graphical abstract

Quantum mechanics/molecular mechanics (QM/MM) simulations are powerful tools for modeling complex molecular systems; however, their predictability has been constrained by the ambiguous definition of the QM region. An electronically informed protocol is introduced that defines QM regions by quantifying guest‐induced orbital shifts and charge‐redistribution, providing transferable and interpretable boundaries across both materials and biological systems.

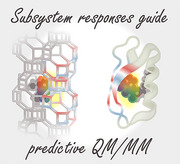

## 1 Introduction

Quantum chemistry has provided deep insights into the electronic structure and reactivity of molecules. For small‐ and medium‐sized systems, advances in density functional theory (DFT), correlated wavefunction methods, and machine learning‐augmented approaches have enabled quantitative predictions of molecular properties, thermodynamics, and even reaction kinetics with chemical accuracy. These developments have transformed our ability to rationalize experiments and to design molecules in areas ranging from homogeneous catalysis to organic electronics [1, 2, 3, 4, 5, 6, 7, 8]. Despite these advances, extending such accuracy to large and complex assemblies—including crystalline solids, polymers, and biomolecular machines—remains a central challenge. The large number of atoms, diverse interactions, and long‐time dynamics render fully quantum‐mechanical treatments impractical, while classical models cannot describe bond rearrangements, charge transfer, or excited‐state processes that often define chemical function.

Hybrid quantum mechanics/molecular mechanics (QM/MM) methods [9] bridge this gap by treating the chemically critical region quantum‐mechanically while modeling the environment with molecular mechanics. This approach has extended quantum accuracy to enzymatic active sites, adsorption centers in solids, and local defects in materials. Over the past decades, QM/MM has become a standard tool in biochemistry [10] and materials science [11], with successes in elucidating enzymatic catalysis [12, 13], drug binding [14, 15], and zeolite reactivity [16, 17]. These accomplishments highlight the versatility of QM/MM. At the same time, the predictive power of QM/MM is still constrained by how the quantum region is defined. Current practice relies on intuition, heuristic cutoffs, or system‐specific conventions, introducing ambiguity and often neglecting long‐range electronic effects essential for accurate energetics and binding affinities [18, 19, 20]. Consequently, QM/MM is frequently confined to retrospective analysis rather than predictive design.

Several strategies—including charge deletion analysis (CDA) [21], charge shift analysis (CSA) [22], and Fukui shift analysis (FSA) [23]—have sought to overcome this limitation by setting a physically appropriate QM region. CDA evaluates the impact of the full MM electronic state environment on the QM region by systematically perturbing MM point charges, which requires a large number of independent QM/MM calculations. CSA directly captures electronic density redistribution using very large, extended QM regions, whereas FSA evaluates changes in frontier‐electron reactivity within reduced QM model systems. These complementary approaches therefore reflect an inherent trade‐off between computational cost and physical completeness. This unmet need motivates the present work: to establish a transferable, computationally lightweight protocol that unifies QM/MM practices across both biological and materials domains.

## 2 Methods

We propose an efficient protocol that defines QM regions from ligand‐induced electronic responses, quantified in a single semiempirical fragment molecular orbital (FMO) calculation, one of the representative fragmentation‐based quantum chemical methods [24, 25, 26, 27, 28, 29]. The framework is lightweight, scalable to systems exceeding ten thousand atoms, and compatible with high‐throughput pipelines. By capturing both local orbital perturbations and long‐range charge‐redistribution effects, it transforms QM/MM into a predictive and design‐oriented approach applicable to both biomolecular assemblies and material systems. Grounding the method in FMO also links it to extensive literature in quantum biochemistry and solid‐state catalysis, providing a natural convergence of insights across disciplines.

### 2.1 Protocol Design

**FIGURE 1:**
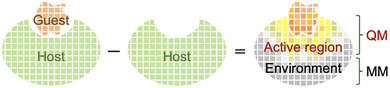
Concept of this study: We quantitatively define QM/MM boundaries by decomposing host–guest electronic responses with linear‐scaling quantum chemical methods (e.g., FMO), using MO‐shift and charge‐redistribution descriptors (see Supporting Information).

Electronic‐response descriptors are extracted from semiempirical FMO‐DFTB (density functional tight binding) calculations [30, 31] in a single computational step (Figure 1):

- MO‐Shift Analysis – monitoring changes in frontier orbital energies of fragments upon guest binding to capture local sensitivity.
- Charge‐Redistribution Analysis – maps fragment charge variations and their flows to reveal nonlocal electronic propagation.

Both descriptors can be efficiently obtained within the FMO framework. FMO naturally partitions complex systems into chemically meaningful fragments, enabling scalable evaluation of monomer‐level (FMO1) and dimer‐level (FMO2) interactions. Together, these descriptors yield both local orbital‐level and nonlocal charge‐redistribution‐level insights, offering a robust and interpretable rule for QM region selection.

## 3 Proof‐of‐Concept Applications

**FIGURE 2:**
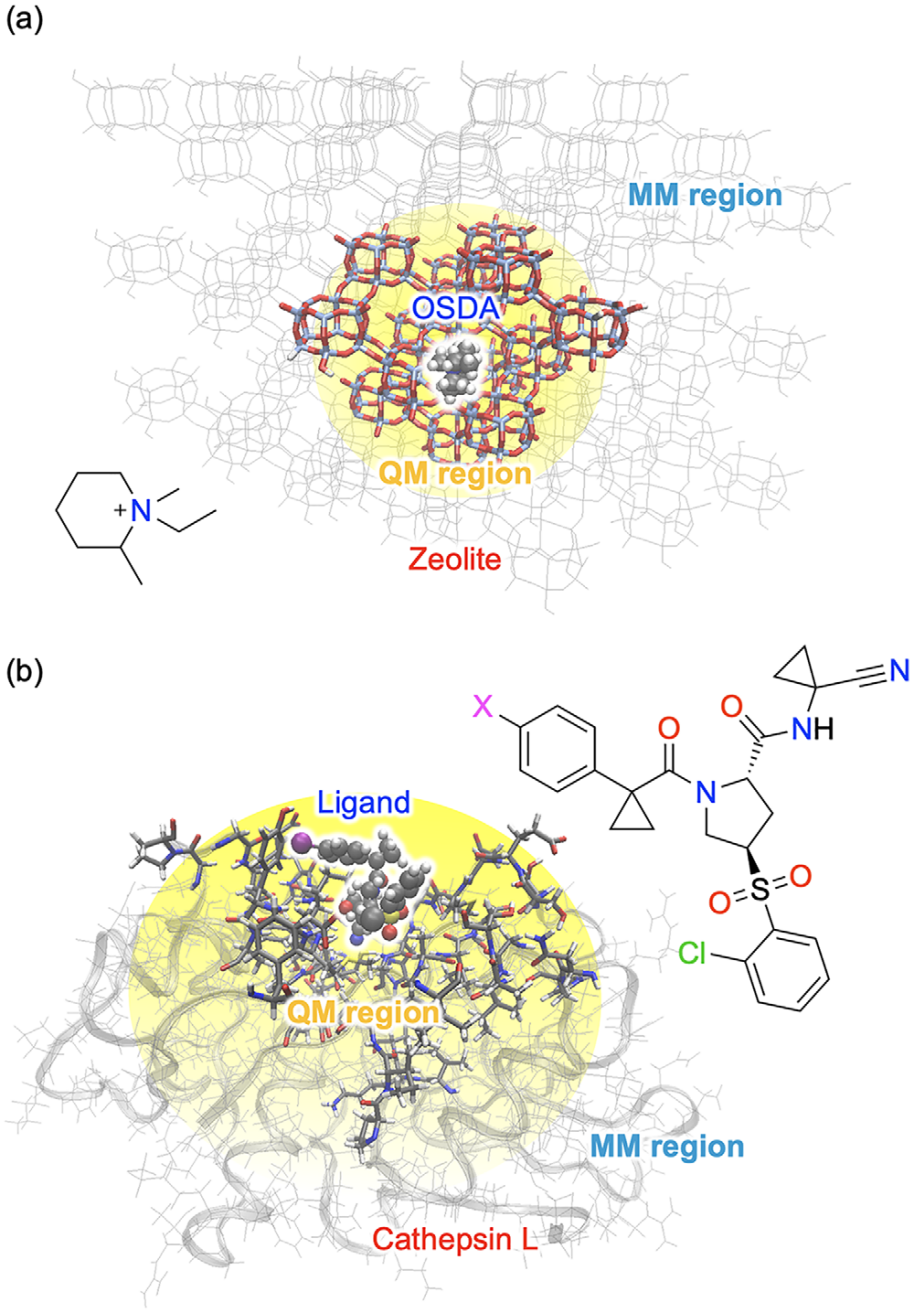
Representative systems used to validate the protocol: (a) CHA‐type zeolite complexed with an OSDA molecule (1‐ethyl‐1,2‐dimethylpiperidinium), and (b) human cathepsin L bound to a small‐molecule inhibitor. Binding sites are highlighted using licorice representation.

We evaluated the protocol in two chemically distinct systems (Figure 2): (i) a CHA‐type zeolite–OSDA (organic structure‐directing agent) complex [32], representing inorganic porous materials with long‐range host–guest coupling, and (ii) a human cathepsin L–inhibitor complex [33], exemplifying enzymatic recognition with charge‐redistribution networks. These testbeds allow evaluation of accuracy, efficiency, and transferability under contrasting regimes.

### 3.1 Inorganic Materials System: Zeolite–OSDA

We first applied our protocol to a CHA‐type zeolite framework complexed with 1‐ethyl‐1,2‐dimethylpiperidinium as an OSDA, a representative system in zeolite synthesis. OSDAs are traditionally regarded as geometric templates that direct framework topology, yet mounting evidence indicates that their functions cannot be explained by sterics alone. Instead, subtle host–guest electronic interactions—charge‐redistribution, orbital polarization, and long‐range electrostatics—govern both framework stability and nucleation kinetics. Accurately capturing these effects requires a QM region that extends beyond the immediate binding pocket.

**FIGURE 3:**
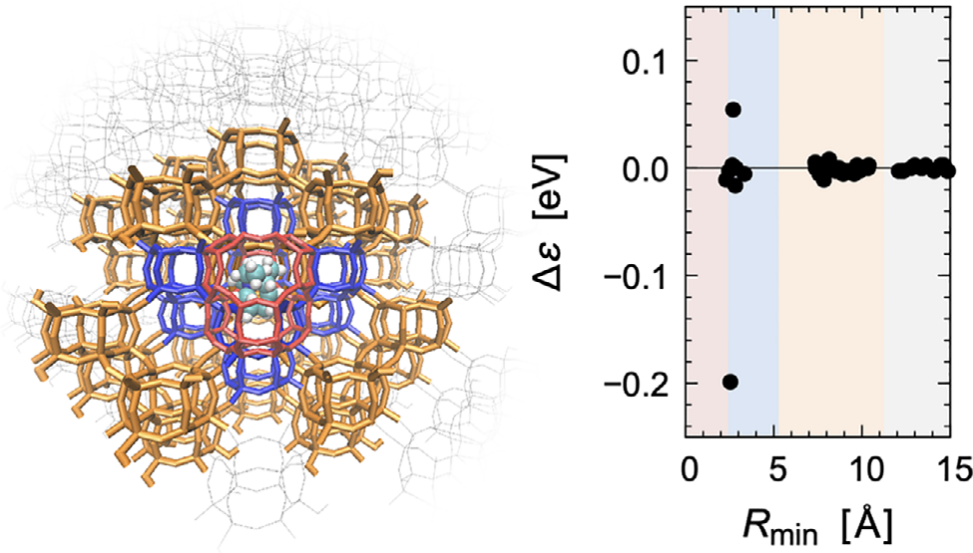
Spatial mapping of MO energy shifts in zeolite framework fragments upon OSDA binding. The horizontal axis indicates the minimum atomic distance from the OSDA (*R*_min_), and the vertical axis shows the change in HOMO‐LUMO gap (Δε) for each zeolite fragment. Red, blue, and yellow correspond to the Empirical [34], Short‐range, and MO‐based models, respectively.

Figure 3 presents the spatial mapping of MO energy shifts across zeolite fragments upon OSDA binding. A systematic decay of orbital perturbations with increasing distance from the OSDA was observed, converging around *R*_min_ ≈ 11 Å, consistent with a second coordination shell (Table S1). This indicates that the guest molecule electronically perturbs not only the binding cavity but also remote framework regions, underscoring the inherently delocalized nature of host–guest coupling in porous solids.

On this basis, we defined the QM region using two different criteria: (i) the conventional Short‐range model (5 Å cutoff, fragments with interaction energy > 2 kcal/mol), and (ii) the MO‐based model (*R*_min_ ≤ 11 Å, as determined by Δε convergence). Several electronic properties obtained from both were compared against the fully quantum mechanical (Whole‐FMO) reference. Table 1 shows that the Short‐range model significantly deviates from the reference in ionization potential (IP) and electron affinity (EA). By contrast, the MO‐based model reproduces all quantities within 0.1 eV, while reducing the computational cost by nearly half of magnitude relative to the Whole‐FMO.

**TABLE 1:** The number of QM atoms, CPU time [min], and the OSDA electronic properties (IP and EA [eV]) for QM/MM models in Figure 3 and the corresponding fully quantum mechanical Whole‐FMO reference.

Model	Empirical	Short‐range	MO‐based	Whole‐FMO
Number of QM atoms	66	366	1326	3286
CPU time	0.5	0.7	2.4	4.5
IP	9.9	10.1	10.2	10.3
EA	3.5	3.4	3.2	3.2

These results carry two important implications. First, long‐range host–guest coupling is indispensable for capturing the correct electronic properties of OSDAs, and thus for understanding their ability to direct framework topology. Second, the MO‐shift criterion provides a general rule for selecting relevant framework fragments, eliminating heuristic assumptions that have historically limited QM/MM studies of zeolites.

Looking forward, such electronically informed partitioning could provide a foundation for rational OSDA design in high‐throughput screening pipelines. By quantifying the electronic reach of candidate OSDAs, one can predict not only their geometric fit but also their capacity to stabilize metastable frameworks, accelerate nucleation, or modulate catalytic acid sites. In this sense, our method offers a design principle that bridges electronic‐structure theory with materials synthesis, paving the way toward predictive, electronically guided discovery of zeolite frameworks.

### 3.2 Biomolecular System: Cathepsin–Inhibitor Complex

We next examined a cathepsin L–inhibitor system, a prototypical enzyme–ligand complex widely studied in drug discovery. Enzymatic active sites are embedded in a dense network of residues, water molecules, and counterions, where subtle electronic perturbations often dictate recognition and catalytic efficiency. Conventional QM/MM studies of such systems have typically relied on heuristic definitions of the binding pocket, but these approaches risk neglecting long‐range coupling and charge‐redistribution effects that underpin allosteric regulation.

**FIGURE 4:**
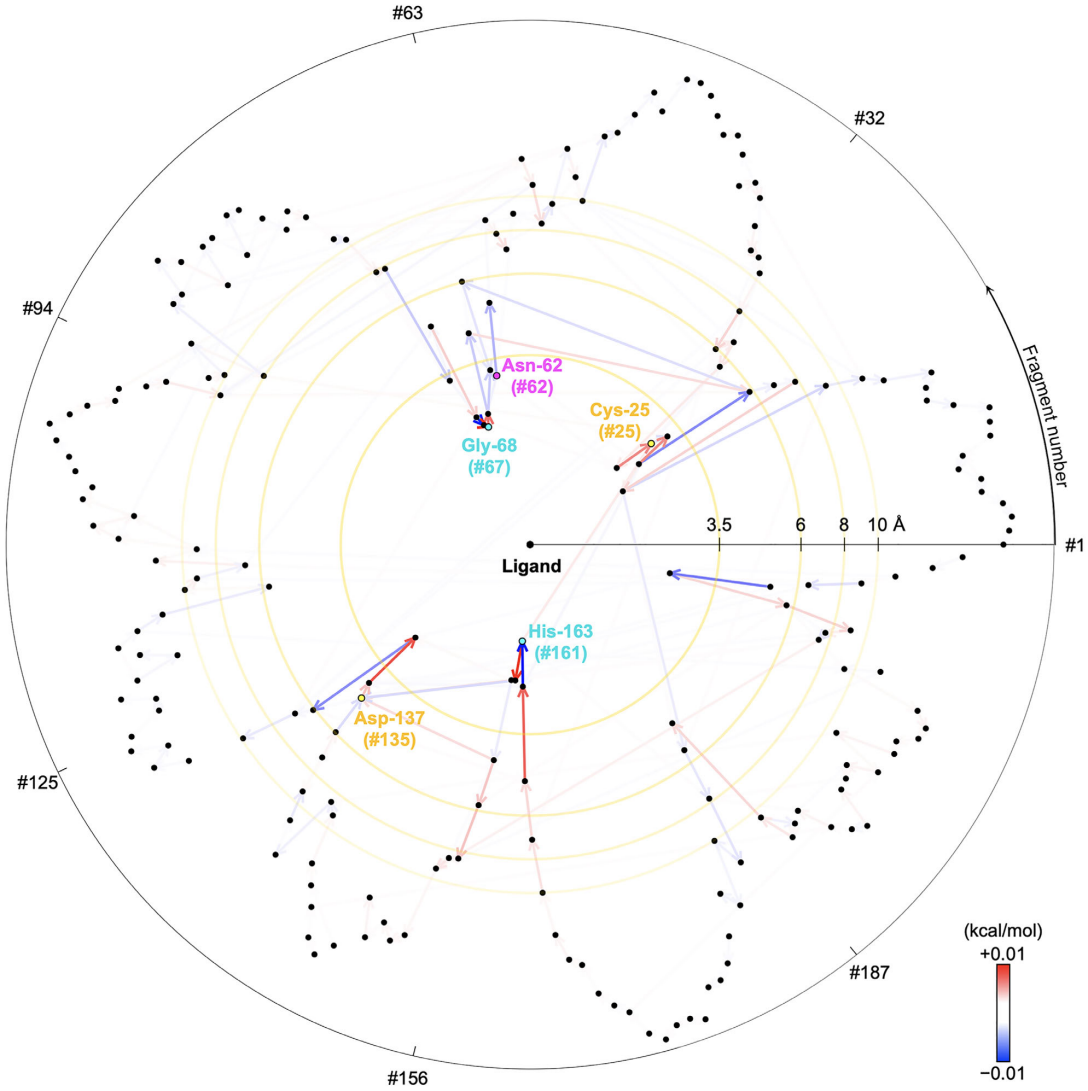
Spatial visualization of the charge‐redistribution network within the human cathepsin L–inhibitor complex (PDB ID: 2YJ8). A black dot represents each amino‐acid fragment. Halogen‐ and hydrogen‐bonded residues are colored magenta and cyan, and “hub” fragments involved in indirect electrostatic mediation are shown in yellow, respectively. The logarithm of the minimum atomic distance from the ligand (log*R*_min_) is shown on the radial axis; the fragment index is mapped angularly. Arrows denote charge transfer direction; blue and red indicate increases and decreases in magnitude, respectively.

Our analysis of MO energy shifts revealed convergence around *R*_min_ ≈ 8 Å of the ligand (Figure S1), consistent with the localized nature of frontier orbital perturbations. In addition, when charge‐redistribution was tracked at the FMO2‐DFTB level, we observed nonlocal propagation of electronic influence across the active‐site network (Figure 4). Notably, Gly‐164 and Asp‐162 were found to be electronically coupled via His‐163, which emerged as a charge‐redistribution hub residue. This demonstrates that the binding pocket is not an isolated unit but part of a dynamic charge‐redistribution network extending across the enzyme.

**FIGURE 5:**
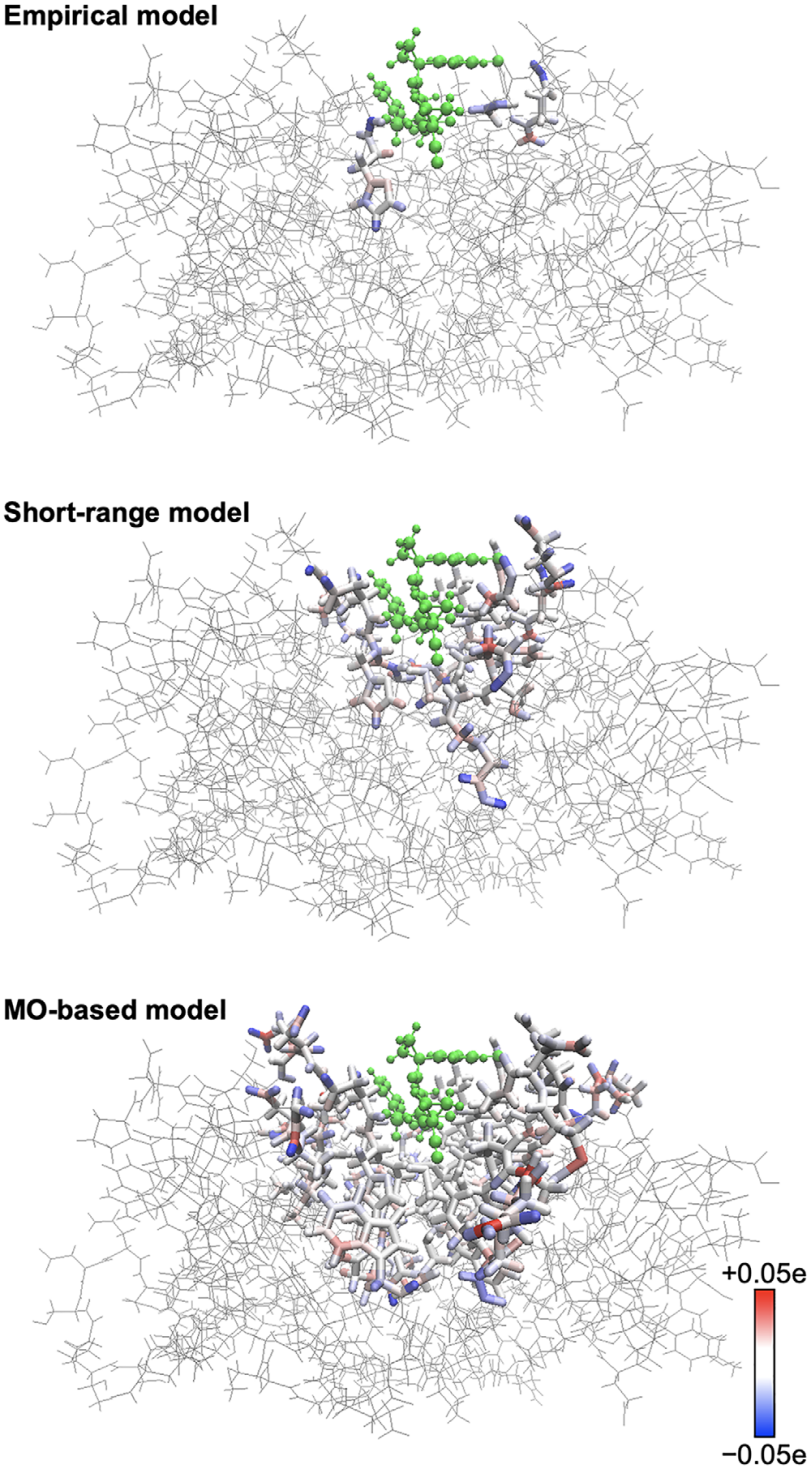
Comparison of atomic charge distribution errors relative to the Whole‐FMO calculation for different QM region models (top: Empirical, middle: Short‐range, bottom: MO‐based models). Blue and red indicate overestimation and underestimation of atomic charge, respectively.

Quantitative comparisons highlight the predictive value of this electronically informed definition. Binding energies obtained with the MO‐based model (–95.6 kcal/mol) closely matched the Whole‐FMO reference (–96.0 kcal/mol), while both the Empirical [33] (–13.9 kcal/mol) and Short‐range (–83.6 kcal/mol) models severely underestimated interactions. Charge density analyses (Figure 5) revealed that the Empirical and Short‐range models introduce artificial localization, whereas the MO‐based model preserves smooth, physically consistent distributions. Pair interaction energy decomposition analysis (PIEDA) [35] (Table 2) further confirmed that Empirical and Short‐range models mainly misrepresent electrostatics and dispersion contributions, while the MO‐based approach closely reproduces the full interaction profile.

**TABLE 2:** PIEDA between the ligand and amino‐acid residues [kcal/mol]. The MO‐based model closely replicates the Whole‐FMO energy components.

	Empirical	Short‐range	MO‐based	Whole‐FMO
Electrostatic	−15.6	−50.9	−61.0	−54.9
Exchange‐repulsion	−1.7	0.4	0.0	0.0
Charge‐transfer	−2.0	−3.2	−2.7	−2.0
Dispersion	−11.4	−76.1	−84.2	−84.2

**FIGURE 6:**
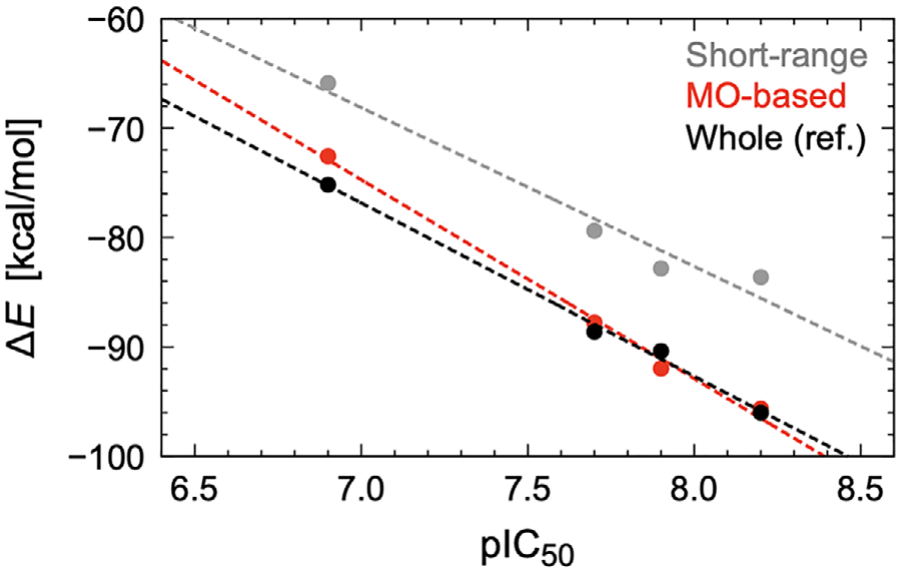
Comparison of binding energy predictions across models. The MO‐based model (red) reproduces the experimental pIC_50_ values [36] (CH_3_: 6.9, Cl: 7.7, Br: 7.9, I: 8.2) with correct rank order and chemical accuracy, outperforming Short‐range definitions (gray).

Most compellingly, the MO‐based QM region reproduced not only absolute binding energies but also experimental structure‐activity relationships for substituted inhibitors (Figure 6 and Table 3). Both rank order and magnitude were captured within ±1–2 kcal/mol, establishing chemical accuracy. This is a level of quantitative fidelity rarely achieved in QM/MM studies of drug–target systems, where manual QM definitions often fail to generalize.

**TABLE 3:** Ligand binding energies [kcal/mol] for different QM region models. Values in parentheses indicate deviations from the Whole‐FMO references.

	Empirical	Short‐range	MO‐based	Whole‐FMO
CH_3_	−8.8	−65.9	−72.6	−75.2
	(66.4)	(9.3)	(2.6)	
Cl	−11.4	−79.4	−87.8	−88.6
	(77.2)	(9.2)	(0.8)	
Br	−14.3	−82.8	−92.0	−90.4
	(76.0)	(7.5)	(‐1.6)	
I	−13.9	−83.6	−95.6	−96.0
	(82.1)	(12.4)	(0.4)	

These results underscore two key insights. First, ligand binding induces electronic perturbations that extend beyond the immediate pocket, propagating through a web of charge‐redistribution residues. This mechanistic view connects QM/MM partitioning with long‐standing concepts of allostery and charge‐redistribution in proteins, providing a physically grounded rationale for why distal residues influence binding and catalysis. Second, the robustness of the MO‐based definition under structural perturbations makes it ideally suited for integration with QM/MM molecular dynamics (QM/MM‐MD). This opens the door to predictive simulations of enzyme–ligand recognition, catalytic turnover, and inhibitor optimization on timescales relevant to drug discovery.

Looking ahead, this approach could transform how we model biomolecular recognition. By anchoring QM/MM boundaries in quantifiable electronic response criteria, rather than static heuristics, it becomes possible to establish transferable protocols across enzyme families, accelerate in silico drug screening, and provide a theoretical basis for designing ligands that exploit or disrupt charge‐redistribution pathways. Such advances would elevate QM/MM from a specialized analysis tool to a predictive design platform in chemical biology and pharmaceutical science.

## 4 Discussion

The representative validation on both zeolites and enzymes demonstrates that electronically informed partitioning is not confined to a single domain, but instead provides a transferable principle that bridges catalysis, materials science, and chemical biology. Moreover, we extended the assessment to two additional classes of host–guest systems—a methane‐hydrate–ammonium‐ion complex and a protein–glycan assembly (*N*‐acetyllactosamine bound to galectin) (Figures S2 and S3). Across these cases, the MO‐based electronic‐response analysis remained quantitatively reliable, further underscoring the generality of the protocol. Our results across both materials and biological systems converge on a unifying principle: guest binding induces electronic responses that extend beyond the immediate interaction site, shaping function through both local and nonlocal channels. By quantifying these responses in a computationally efficient manner, the proposed protocol provides a transferable design rule for QM/MM region selection. These validations are particularly significant because prior reviews have highlighted the siloed nature of QM/MM practice in biology vs. materials science [10, 11]. By demonstrating robustness across such disparate systems, our protocol establishes electronically informed partitioning as a cross‐domain standard that unifies best practices in both materials and biomolecular modeling.

In materials science, this offers a route toward predictive design of framework‐directing agents, enabling the electronic‐level tailoring of zeolite synthesis and catalysis. In biochemistry, it establishes a foundation for predictive enzyme–ligand modeling, with implications for drug discovery pipelines that demand both accuracy and scalability. More broadly, the approach demonstrates how fragment‐based electronic descriptors can bridge the divide between localized quantum events and macroscopic function, potentially inspiring future integrations with machine learning and multiscale modeling. These results suggest that electronically informed QM/MM partitioning may serve as a generalizable design principle, positioning QM/MM as not only a mechanistic tool but also a predictive framework for molecular engineering.

While the present validation was performed at the semiempirical DFTB/MM level, the electronically informed definition is inherently transferable to higher‐level Hamiltonians. Because the cost of full quantum references grows steeply with system size, the acceleration afforded by our protocol becomes even more pronounced at the DFT/MM and ab initio/MM levels. In fact, preliminary internal tests already indicate relative speed‐ups substantially beyond the one order of magnitude demonstrated here, suggesting that two or more orders of magnitude can be realistically attained. This scalability underscores the potential of electronically informed partitioning to extend predictive QM/MM modeling into regimes of accuracy and system size previously regarded as impractical. Taken together, these results position electronically informed partitioning as a practical, Hamiltonian‐agnostic rule that scales from DFTB/MM to DFT/MM and ab initio/MM without re‐tuning system‐specific heuristics. We note that the present benchmarks focus on ground‐state properties; extending the electronically informed partitioning to excited‐state and reactive dynamics (e.g., analysis of the activation barriers for bio‐ and material‐based catalytic systems) will be an important next step.

## 5 Conclusion

We have developed a physically interpretable protocol for defining active QM regions in hybrid QM/MM simulations by quantifying ligand‐induced MO energy shifts and fragment charge redistribution through a single semiempirical FMO calculation. This low‐cost analysis enables accurate and transferable QM region identification that remains robust under structural fluctuations and compatible with higher‐level Hamiltonians such as FMO‐DFT or MP2.

By grounding QM/MM boundaries in quantifiable electronic responses, the method transforms QM/MM from a retrospective tool into a predictive framework applicable across materials and biomolecular systems. It further provides a priori guidance when the active site is unknown, reducing empirical trial‐and‐error, narrowing the design space, and enabling more targeted ligand optimization. Beyond its immediate utility, the approach offers an electronically grounded foundation for multiscale modeling and opens a route toward chemistry by prediction rather than explanation, positioning QM/MM as a versatile paradigm for predictive molecular engineering.

## 6 Computational Details

All calculations were performed within the FMO framework implemented in GAMESS [25, 37] interfaced with the TINKER package [38].

For zeolite systems, the CHA‐type framework [39] was partitioned into chemically intuitive fragments, and the MM environment was described with the MM3 force field [40]. The quantum region was treated using DFTB with the matsci parameter set [41], with dispersion corrected by D3(BJ) [42].

For enzyme–substituted inhibitor (CH_3_, Cl, Br, and I) complexes, initial coordinates were extracted from each crystal structure (PDB IDs: 2XU5, 2YJC, 2YJ2, 2YJ8) [33, 36]. Protonation states were assigned at optimum pH 5.5, the system was neutralized with sodium counterions, and solvated in a water sphere of 33 Å radius. Geometry optimization was performed using the AMBER10:EHT force field, and structural modeling was conducted with the MOE software [43]. Using the optimized geometry of each complex, protein fragments corresponded to individual amino‐acid residues. Ligands were parameterized with GAFF [44], and the AMBER ff99 force field [45] was used for proteins and solvent. DFTB with dispersion corrected by D3(BJ) calculations used the 3OB(XB) parameter set [46], PIEDA was performed to separate electrostatic, exchange‐repulsion, charge‐transfer, and dispersion contributions [35].

Solvent effects were consistently evaluated by employing the PCM (polarizable continuum model) [47] and MM‐GBSA (molecular mechanics with generalized Born surface area) [48] models for both zeolite and cathepsin systems, allowing us to assess the role of dielectric screening on host–guest and protein–ligand interactions.

For the FMO calculations on the protein–ligand complex, the macromolecule was partitioned into chemically meaningful units—namely, individual amino‐acid residues (and monosaccharide motifs). Fragmentation followed the established protocol in which C─C single bonds, of which electronic density is predominantly localized, are cleaved while preserving the conjugated framework. This scheme enables the orbitals of each fragment to faithfully reproduce those obtained from conventional QM calculations, thereby minimizing artifacts associated with fragmentation.

## Conflicts of Interest

The authors declare no conflict of interest.

## Supporting information




**Supporting File**: advs73483‐sup‐0001‐SuppMat.pdf.

## Data Availability

The data that support the findings of this study are available in the supplementary material of this article.
