# Supplementary material for: Ligand‐Induced Electronic Response Enables Predictive QM/MM Simulations
Source: Adv Sci (Weinh). 2025 Dec 22;13(17):e19137. doi: 10.1002/advs.202519137 (PMC13042439; doi:10.1002/advs.202519137)
Supplement: Supplementary file 1 — Supporting File: advs73483‐sup‐0001‐SuppMat.pdf. [file ADVS-13-e19137-s001.pdf]

Supporting Information

Ligand-Induced Electronic Response Enables Predictive QM/MM Simulations

*Nichika Ozawa, Nahoko Kuroki, Hirotoshi Mori**

Nichika Ozawa†
Department of Applied Chemistry, Faculty of Science and Engineering, Chuo University,
1-13-27 Kasuga, Bunkyo-ku, Tokyo 112-8551, Japan

Nahoko Kuroki
Faculty of Core Research, Natural Science Division, Ochanomizu University,
2-1-1 Otsuka, Bunkyo-ku, Tokyo 112-8610, Japan

Hirotoshi Mori
Department of Applied Chemistry, Faculty of Science and Engineering, Chuo University,
1-13-27 Kasuga, Bunkyo-ku, Tokyo 112-8551, Japan
E-mail: qc-forest.19d@g.chuo-u.ac.jp

†Present address; Department of Chemistry and Biochemistry, Graduate School of Humanities and Sciences, Ochanomizu University, 2-1-1 Otsuka, Bunkyo-ku, Tokyo 112-8610, Japan

Table of Contents

S1. Theoretical Overview of the Fragment Molecular Orbital (FMO) Method
S2. Preparation of TINKER Parameters for the Ligand
S3. Supporting Tables
S4. Supporting Figures
S5. Computational Details for Validations
S6. Supporting References

S1. Theoretical Overview of the Fragment Molecular Orbital (FMO) Method

The Fragment Molecular Orbital (FMO) method partitions a large molecular system into chemically meaningful fragments and reconstructs the total electronic energy from monomer and dimer terms:

$$E_{\mathrm{FMO}} = \sum_{I} E'_I + \sum_{I>J} \Delta E_{IJ} + \cdots,$$

where E'_I is the monomer energy of fragment *I* in the electrostatic field of all other fragments, and $\Delta E_{IJ}\left(= E'_{IJ} - E'_I - E'_J\right)$ is the pair interaction energy between fragments *I* and *J*. This expansion naturally incorporates both local electronic structure and inter-fragment interactions with reduced computational cost. Covalent bonds between fragments are handled by hybrid orbital projection schemes, allowing FMO to be applied to proteins, nucleic acids, and extended frameworks. In fragment division, conjugated systems should be preserved, and cleavage should be performed at single bonds where electron distribution is local.

Importantly, the FMO framework is compatible with a wide variety of quantum-chemical Hamiltonians. In addition, Hartree-Fock,[S1] semiempirical[S2], density functional theories (DFTs),[S3,S4] and correlated wavefunction methods such as Møller-Plesset perturbation[S5,S6] theory and coupled cluster theory[S7,S8] have been successfully implemented within FMO, thereby enabling systematic inclusion of electron correlation in large-scale systems.

The method has been extended to hybrid QM/MM frameworks (FMO/MM),[S9] where the FMO region is embedded in a molecular mechanics (MM) environment, as well as to molecular dynamics simulations (FMO-MD),[S10-S14] enabling statistical sampling of fluctuating environments and direct analysis of electronic fluctuations.

In the present study, we leverage this theoretical foundation to establish a seamless protocol for defining QM regions in QM/MM simulations based on ligand-induced electronic responses. In MO-shift analysis, changes in frontier orbital energies of fragments upon guest binding $\Delta\varepsilon\left(= \varepsilon_{\mathrm{holo}} - \varepsilon_{\mathrm{apo}}\right)$ are calculated. Furthermore, in charge-redistribution analysis, changes in charge-transfer between each fragment upon guest binding $\Delta Q_{I\leftarrow J}\left(= Q_{I\leftarrow J,\mathrm{holo}} - Q_{I\leftarrow J,\mathrm{apo}}\right)$ are tracked; the charge transferred from fragment *J* to *I* is $Q_{I\leftarrow J}$.

These descriptors, obtained in a single semiempirical FMO calculation and that achieve speedy definition of QM region.

S2. Preparation of TINKER Parameters for the Ligand

The procedure for generating TINKER parameters of the ligand applied to the MM region in FMO/MM calculations was adapted from the Amber manual[S15]:

1. The PDB coordinates of the ligand were converted to mol2 format with AM1-BCC charges using Antechamber.
   ```
   antechamber -i lig.pdb -fi pdb -o lig.mol2 -fo mol2 -c bcc -nc 0
   ```

2. A Gaussian input file was generated from the mol2 file, and RESP charges were calculated at the HF/CEP-31G* level with PCM solvent treatment using Gaussian09.

3. A RESP-refined mol2 file was created and missing parameters were supplemented with parmchk2.

4. Amber topology and coordinate files were generated with tleap using GAFF parameters: (source leaprc.gaff)
   ```
   loadamberparams lig_resp.frcmod
   mymol = loadmol2 lig_resp.mol2
   saveamberparm mymol lig.prmtop lig.inpcrd
   quit
   ```

5. The Amber parameter files were converted to TINKER format using a Python script (generate_TINKER_parameters.py, available on GitHub[S16]).

6. Bond connectivity and van der Waals parameters were manually adjusted where necessary.

S3. Supporting Tables

Table S1. MO-shifts ($\Delta\varepsilon_{\mathrm{HOMO}} = \varepsilon_{\mathrm{HOMO,holo}} - \varepsilon_{\mathrm{HOMO,apo}}$ and $\Delta\varepsilon_{\mathrm{LUMO}} = \varepsilon_{\mathrm{LUMO,holo}} - \varepsilon_{\mathrm{LUMO,apo}}$ [eV]) and changes in HOMO-LUMO gap ($\Delta\varepsilon$ [eV]) in zeolite framework fragments upon OSDA binding and minimum atomic distance from the OSDA (R_{min} [Å]).

R_{min}	$\Delta\varepsilon_{\mathrm{HOMO}}$	$\Delta\varepsilon_{\mathrm{LUMO}}$	$\Delta\varepsilon$	R_{min}	$\Delta\varepsilon_{\mathrm{HOMO}}$	$\Delta\varepsilon_{\mathrm{LUMO}}$	$\Delta\varepsilon$	R_{min}	$\Delta\varepsilon_{\mathrm{HOMO}}$	$\Delta\varepsilon_{\mathrm{LUMO}}$	$\Delta\varepsilon$
2.3	−3.04	−3.33	−0.01	9.9	−0.30	−0.37	0.00	18.6	−0.22	−0.22	0.00
2.5	−0.96	−1.04	0.00	10.1	−0.30	−0.30	0.00	18.6	−0.15	−0.15	0.00
2.5	−3.33	−8.74	−0.20	10.2	−0.30	−0.30	0.00	18.6	−0.22	−0.15	0.00
2.7	−1.55	−1.48	0.00	10.3	−0.44	−0.44	0.00	18.7	−0.22	−0.22	0.00
2.7	−6.89	−5.41	0.05	10.4	−0.37	−0.30	0.00	18.9	−0.22	−0.22	0.00
2.8	−2.67	−3.11	−0.02	12.1	−0.22	−0.30	0.00	18.9	−0.22	−0.22	0.00
2.9	−0.81	−0.81	0.00	12.3	−0.30	−0.37	0.00	19.2	−0.15	−0.22	0.00
3.3	−0.96	−1.11	−0.01	12.3	−0.22	−0.30	0.00	19.2	−0.22	−0.22	0.00
7.4	−0.44	−0.44	0.00	12.8	−0.30	−0.30	0.00	19.9	−0.22	−0.22	0.00
7.4	−0.52	−0.37	0.01	13.0	−0.30	−0.22	0.00	20.1	−0.22	−0.22	0.00
7.6	−0.30	−0.44	−0.01	13.3	−0.22	−0.22	0.00	20.6	−0.15	−0.22	0.00
7.6	−0.44	−0.37	0.00	13.4	−0.22	−0.22	0.00	21.8	−0.22	−0.22	0.00
7.8	−0.30	−0.59	−0.01	13.6	−0.30	−0.22	0.00	22.0	−0.22	−0.22	0.00
8.0	−0.37	−0.37	0.00	14.1	−0.22	−0.30	0.00	22.0	−0.15	−0.22	0.00
8.0	−0.44	−0.30	0.01	14.5	−0.30	−0.22	0.00	23.9	−0.15	−0.15	0.00
8.0	−0.37	−0.37	0.00	14.6	−0.30	−0.22	0.00	24.5	−0.15	−0.15	0.00
8.1	−0.52	−0.30	0.01	14.9	−0.22	−0.30	0.00	24.5	−0.15	−0.15	0.00
8.5	−0.30	−0.37	0.00	14.9	−0.22	−0.30	0.00	24.8	−0.15	−0.15	0.00
8.6	−0.30	−0.30	0.00	15.0	−0.22	−0.22	0.00	25.1	−0.15	−0.15	0.00
8.7	−0.30	−0.30	0.00	15.9	−0.15	−0.15	0.00	25.4	−0.22	−0.15	0.00
8.7	−0.30	−0.37	0.00	16.1	−0.22	−0.22	0.00	26.3	−0.15	−0.15	0.00
8.9	−0.30	−0.44	−0.01	16.3	−0.22	−0.22	0.00	26.7	−0.22	−0.15	0.00
9.2	−0.37	−0.44	0.00	16.9	−0.22	−0.22	0.00	26.8	−0.15	−0.15	0.00
9.5	−0.30	−0.59	−0.01	17.4	−0.22	−0.22	0.00	27.7	−0.15	−0.15	0.00
9.6	−0.30	−0.44	−0.01	17.8	−0.22	−0.22	0.00	27.9	−0.15	−0.15	0.00
9.7	−0.44	−0.44	0.00	18.1	−0.30	−0.22	0.00	29.2	−0.15	−0.07	0.00
9.7	−0.30	−0.22	0.00	18.6	−0.15	−0.22	0.00	34.2	−0.15	−0.15	0.00

S4. Supporting Figures

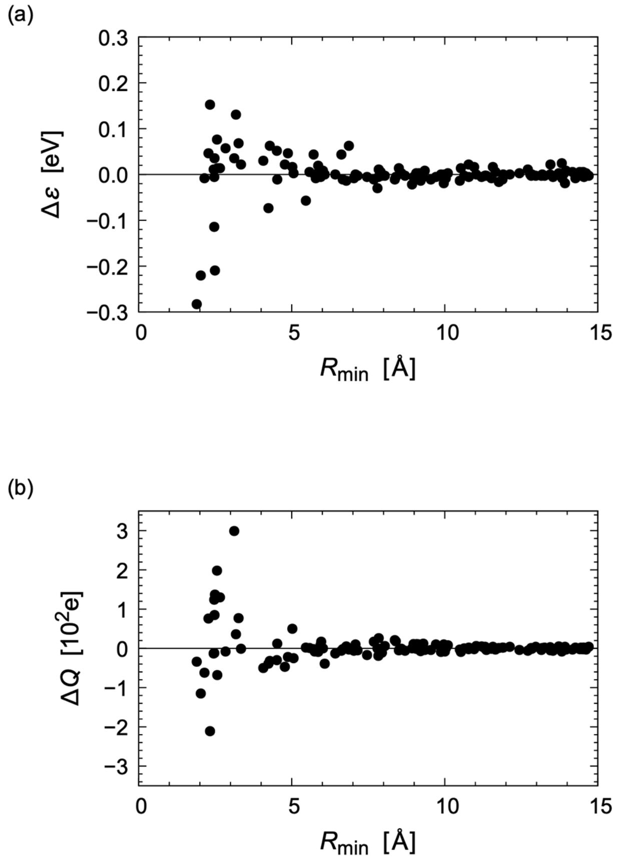

Figure S1. Electronic responses of amino-acid residues upon ligand binding as a function of the shortest atomic distance from the ligand (R_{min}). (a) MO energy response, shown as the change in HOMO-LUMO gap ($\Delta\varepsilon$). (b) Charge-redistribution (ΔQ_I), defined as the difference in net charge before and after ligand binding at the FMO2-DFTB level. The concept of charge-redistribution can be further detailed as follows: the amount of charge transfer of fragment I is given by the sum of the charge donated to fragment J ($\neq I$) and the charge received from fragment J ($\neq I$). If the charge transferred from fragment J to I is $Q_{I\leftarrow J}$ and the total charge-redistribution at fragment I is Q_I, then the change in charge-redistribution upon ligand binding can be expressed as $\Delta Q_I = Q_{I,\mathrm{holo}} - Q_{I,\mathrm{apo}} = \sum_J Q_{I\leftarrow J,\mathrm{holo}} - \sum_J Q_{I\leftarrow J,\mathrm{apo}}$.

(a)

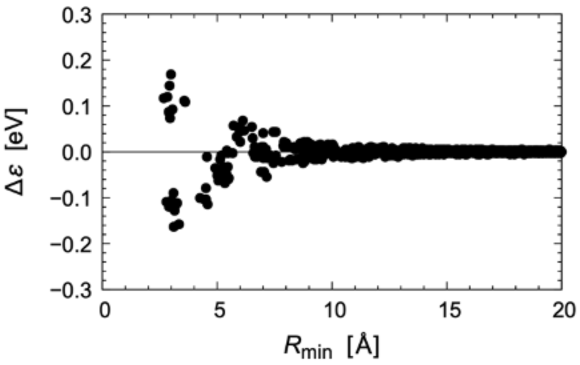

(b)

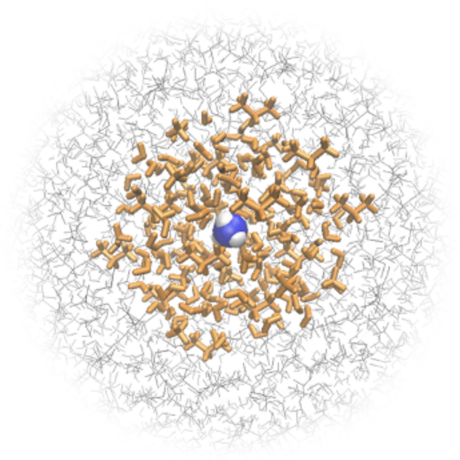

Figure S2. The validation in methane-hydrate–ammonium-ion complex.

(a) Electronic responses (changes in HOMO-LUMO gap (Δε)) of methane-hydrate molecules upon ammonium ion induce as a function of the shortest atomic distance from the ion molecule (R_{min}).

(b) The model of methane-hydrate and an ammonium ion complex. The MO-based model (yellow) can evaluate electronic properties of ammonium ion (central molecule shown in vdW style) accurately respective in Whole-FMO (gray) (IP / EA[eV]: 17.7 / 0.7 in MO-based model, 17.8 / 0.6 in Whole-FMO reference).

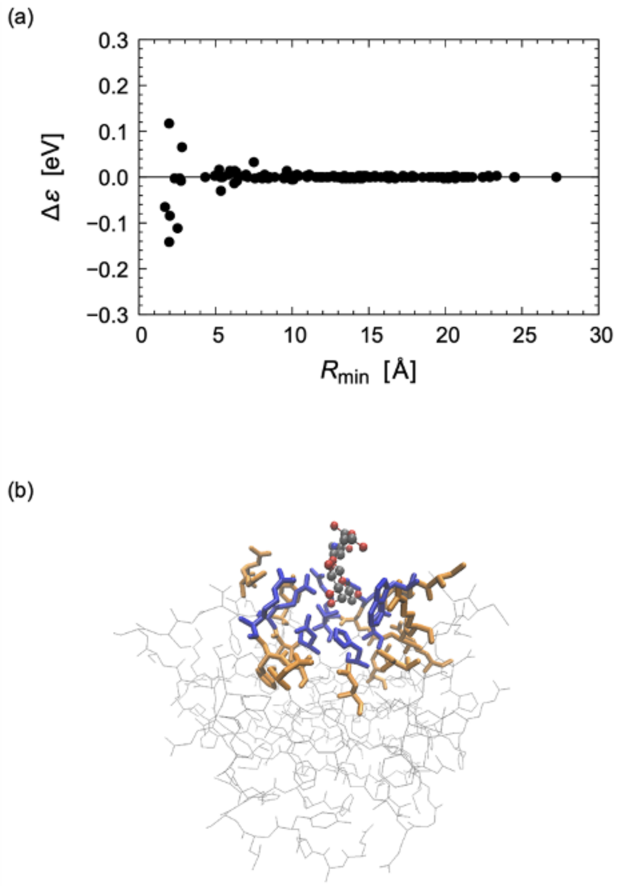

Figure S3. The validation in galectin–*N*-acetyllactosamine complex.

(a) Electronic responses (changes in HOMO-LUMO gap ($\Delta\varepsilon$)) of amino-acid residues upon glycan binding as a function of the shortest atomic distance from the glycan (R_{min}).

(b) The model of galectin–*N*-acetyllactosamine complex. The binding energy was evaluated –31.0 kcal/mol in Short-range model (blue), –19.7 kcal/mol in MO-based model (yellow), and –19.8 kcal/mol in Whole-FMO reference (gray) respectively.

S5. Computational Details for Validations

To further demonstrate the breadth and robustness of our protocol, we performed two additional validations using (i) a methane-hydrate–ammonium-ion complex and (ii) a protein–glycan complex (*N*-acetyllactosamine bound to galectin). These systems were selected to cover a wide spectrum of chemical environments, from rigid molecular assemblies to flexible biomolecular interfaces.

For the methane-hydrate system, the structural model was constructed based on the reference structure,[S17] in which the central methane molecule was replaced with an ammonium ion. Each constituent molecule was treated as an individual fragment.

For the protein–glycan complex, initial coordinates were obtained from the crystal structure (PDB ID: 2YY1). Protonation states were assigned assuming physiological pH (pH 7.0). The protein was fragmented at the amino-acid residue level, whereas the glycan moiety was partitioned into monosaccharide fragments.

All FMO calculations were carried out using the GAMESS program with the 3OB parameter set,[S18] along with D3(BJ) dispersion corrections.[S19] Solvent effects were consistently incorporated using the polarizable continuum model (PCM).[S20]

S6. Supporting References

S1. K. Kitaura, E. Ikeo, T. Asada, T. Nakano, and M. Uebayasi, "Fragment Molecular Orbital Method: An Approximate Computational Method for Large Molecules," *Chemical Physics Letters* **1999**, *313*, 701–706. https://doi.org/10.1016/S0009-2614(99)00874-X

S2. Y. Nishimoto, D. G. Fedorov, and S. Irle, "Density-Functional Tight-Binding Combined with the Fragment Molecular Orbital Method," *Journal of Chemical Theory and Computation* **2014**, *10*, 4801–4812. https://doi.org/10.1021/ct500489d

S3. D. G. Fedorov and K. Kitaura, "On the Accuracy of the 3-Body Fragment Molecular Orbital Method (FMO) Applied to Density Functional Theory," *Chemical Physics Letters*, **2004**, 389, 129–134. https://doi.org/10.1016/j.cplett.2004.03.072

S4. Y. Shimodo, K. Morihashi, and T. Nakano, "Examination of Numerical Accuracy on Fragment-DFT Calculations with Integral Values of Total Electron Density Functions," *Journal of Molecular Structure: THEOCHEM*, **2006**, 770, 163–168. https://doi.org/10.1016/j.theochem.2006.05.056

S5. D. G. Fedorov and K. Kitaura, "Second Order Møller-Plesset Perturbation Theory Based upon the Fragment Molecular Orbital Method," *The Journal of Chemical Physics* **2004**, *121*, 2483–2490. https://doi.org/10.1063/1.1769362

S6. Y. Mochizuki, K. Yamashita, T. Murase, *et al.*, "Large Scale FMO-MP2 Calculations on a Massively Parallel-Vector Computer," *Chemical Physics Letters* **2008**, *457*, 396–403. https://doi.org/10.1016/j.cplett.2008.03.090

S7. D. G. Fedorov and K. Kitaura, "Coupled-Cluster Theory Based upon the Fragment Molecular-Orbital Method," *The Journal of Chemical Physics* **2005**, *123*, 134103: 1–11. https://doi.org/10.1063/1.2007588

S8. Y. Mochizuki, K. Yamashita, T. Nakano, *et al.*, "Higher-Order Correlated Calculations Based on Fragment Molecular Orbital Scheme," *Theoretical Chemistry Accounts* **2011**, *130*, 515–530. https://doi.org/10.1007/s00214-011-1036-3

S9. D. G. Fedorov, N. Asada, I. Nakanishi, and K. Kitaura, "The Use of Many-Body Expansions and Geometry Optimizations in Fragment-Based Methods," *Accounts of Chemical Research* **2014**, *47*, 2846–2856. https://doi.org/10.1021/ar500224r

S10. Y. Komeiji, T. Nakano, K. Fukuzawa, *et al.*, "Fragment Molecular Orbital Method: Application to Molecular Dynamics Simulation, 'Ab Initio FMO-MD'," *Chemical Physics Letters* **2003**, *372*, 342–347. https://doi.org/10.1016/S0009-2614(03)00430-5

S11. T. Fujiwara, Y. Mochizuki, Y. Komeiji, *et al.*, "Fragment Molecular Orbital-Based Molecular Dynamics (FMO-MD) Simulations on Hydrated Zn(II) Ion," *Chemical Physics Letters* **2010**, *490*, 41–45. https://doi.org/10.1016/j.cplett.2010.03.020

S12. A. Matsuda and H. Mori, "Theoretical Study on the Hydration Structure of Divalent Radium Ion Using Fragment Molecular Orbital–Molecular Dynamics (FMO–MD) Simulation," *Journal of Solution Chemistry* **2014**, *43*, 1669–1675. https://doi.org/10.1007/s10953-014-0235-7

S13. Y. Kato, T. Fujiwara, Y. Komeiji, *et al.*, "Fragment Molecular Orbital-Based Molecular Dynamics (FMO-MD) Simulations on Hydrated Cu(II) Ion," *Chem-Bio Informatics Journal* **2014**, *14*, 1–13. https://doi.org/10.1273/cbij.14.1

S14. H. Mori, N. Hirayama, Y. Komeiji, and Y. Mochizuki, "Differences in Hydration between *cis*- and *trans*-Platin: Quantum Insights by *Ab Initio* Fragment Molecular Orbital-Based Molecular Dynamics (FMO-MD)," *Computational and Theoretical Chemistry* **2012**, *986*, 30–34. https://doi.org/10.1016/j.comptc.2012.02.008

S15. *Amber 2024 Reference Manual,* https://ambermd.org/doc12/Amber24.pdf (accessed Sep 27, 2025).

S16. E. Leddin, *emleddin/pdbxyz-xyzpdb,* https://github.com/emleddin/pdbxyz-xyzpdb (accessed Sep 27, 2025).

S17. Q. Guo, H.-C. Wang, X.-Y. Liu, *et al*., "Computational Analysis of Vibrational Spectra of Hydrogen Bonds in sII and sH Gas Hydrates," *ACS Omega* **2023**, *8*, 11634–11639. https://doi.org/10.1021/acsomega.3c01237

S18. M. Gaus, A. Goez, and M. Elstner, “Parametrization and Benchmark of DFTB3 for Organic Molecules,” *Journal of Chemical Theory and Computation* **2013**, *9*, 338–354. https://dx.doi.org/10.1021/ct300849w

S19. S. Grimme, A. Hansen, J. G. Brandenburg, and C. Bannwarth, “Dispersion-Corrected Mean-Field Electronic Structure Methods,” *Chemical Reviews* **2016**, *116*, 5105–5154. https://doi.org/10.1021/acs.chemrev.5b00533

S20. D. G. Fedorov, K. Kitaura, H. Li, J. H. Jensen, and M. S. Gordon, “The Polarizable Continuum Model (PCM) Interfaced with the Fragment Molecular Orbital Method (FMO),” *Journal of Computational Chemistry* **2006**, *27*, 976–985. https://doi.org/10.1002/jcc.20406
